# Lived experience of gaming disorder among people with psychotic disorders: implications for tailored interventions and clinical management

**DOI:** 10.1097/YCO.0000000000001013

**Published:** 2025-05-23

**Authors:** Maxime Huot-Lavoie, Laurent Béchard, Olivier Corbeil, Olivier Roy, Sophie L’Heureux, Ingrid Salvini, Catherine Lehoux, Anne-Marie Essiambre, Chantale Thériault, Sébastien Brodeur, Marie-France Demers, Yasser Khazaal, Marc-André Roy

**Affiliations:** a Faculty of Medicine; b CERVO Brain Research Centre, Université Laval; c Clinique Notre-Dame des Victoires, Centre intégré universitaire en santé et services sociaux de la Capitale Nationale; d Faculty of Pharmacy; e Faculty of Nursing; f École de psychologie, Faculté des sciences sociales, Université Laval; g Child and adolescent psychiatry department, Hôtel-Dieu de Lévis, Centre intégré en santé et services sociaux de Chaudière-Appalaches, Québec, Canada; h Department of Psychiatry, Lausanne University; i Lausanne University Hospital Research Center, Lausanne, Switzerland

**Keywords:** digital well being, early intervention, Gaming Disorder, lived experiences, psychotic disorders

## Abstract

**Purpose of review:**

Despite growing recognition of the impact of Gaming Disorder in individuals with psychotic disorders, little is known about the clinical and personal implications of this dual diagnosis. Preliminary data suggest that Gaming Disorder may be associated with increased psychotic symptoms and reduced occupational and social functioning. However, insight from lived experience remain largely absent, despite their importance.

**Recent findings:**

This review synthesizes recent literature on the comorbidity between Gaming Disorder and psychotic disorders, highlighting the scarcity of research in this emerging field. It also presents preliminary findings from an ongoing qualitative study focussing on the lived experiences of individuals receiving early psychosis intervention. These data focus on participants’ motivations for gaming and their perceptions of both positive and negative effects gaming has on their life.

**Summary:**

This review underscores the significant lack of data on the dual diagnosis of Gaming Disorder and psychosis. Early qualitative insights reveal diverse gaming motivations, including symptom regulation, anxiety management, cognitive stimulation, and social connection. These first-person accounts emphasize the functional role of gaming and the need for recovery-oriented care. Integrating lived experience into research and clinical practice can improve relevance, support nuanced interventions, and advance our understanding of behavioral addictions in early psychosis.

## INTRODUCTION

Psychotic disorders affect up to 3% of the population [1], with antipsychotic medications leading to remission of positive symptoms in approximately 80% of individuals within the first year of treatment [2,3]. However, when broader recovery measures —encompassing autonomy, meaningful social roles, and life satisfaction— are considered, only about a third of individuals with first-episode psychosis (FEP) achieve full recovery [4^▪▪^].

A growing body of literature highlights the impact of comorbidities on recovery [5–9], particularly substance use disorders (SUDs), which are prevalent in FEP and consistently associated with earlier onset [10–12], more severe symptoms [12–18], social and occupational impairments [15,17,19,20], and higher relapse and disengagement rates [5,21–27].

As the field increasingly recognizes behavioral addictions, Gaming Disorder (GD) has emerged as a relevant comorbidity in FEP. GD was officially recognized during the World Health Assembly in May 2019 and defined in the 11^th^ edition of the International Classification of Diseases (ICD-11) as an impaired control over gaming, increased priority given to gaming, and continued gaming despite negative consequence [28].

The prevalence of GD is estimated at 3.3% in the general population [29]. However, individuals experiencing a psychotic episode may be at higher risk for developing GD. Both conditions often emerge during adolescence or early adulthood, disproportionately affect males and are associated with social withdrawal and comorbid anxiety disorders [30–33]. A recent scoping review noted that the existing literature on the co-occurrence of GD and psychosis is limited primarily to case reports and cross-sectional studies, precluding conclusions about causality or temporality [34^▪^]. Some reports describe psychotic episodes arising during periods of increased gaming in young adults [35,36], while other report psychosis following abrupt cessation of excessive gaming [37,38]. This dual pattern resembles that seen in substance use disorders, where both intensive use and withdrawal may precipitate psychotic symptoms [10–12].

Since the publication of the scoping review, an additional 138 papers have been identified using the same methodology. Among these, only five case reports describing a total of ten cases have focussed on the co-presentation of GD and psychotic disorders. A recent case series by Ricci *et al.* described two individuals presenting with both psychotic symptoms and GD, in whom antipsychotic treatment led to symptomatic improvement in both conditions [39^▪^]. Other case reports further support the initial observations of the scoping review: some individuals experienced psychotic episodes following intensive gaming sessions [40^▪^–42^▪^], while others developed symptoms after the abrupt cessation of excessive gaming behavior [42^▪^].

Recent literature also indicates that gaming may confer certain benefits for individuals with psychotic disorders. Therapeutic video games have been associated with improvements in cognitive and social functioning among individuals with psychotic disorders [43–45]. One study suggests that video game use may alleviate self-stigma-related symptoms in individuals with psychosis [46]. A recent case report highlighted the potential use of video games as a coping strategy to reduce anxiety associated with persecutory delusions, while fostering a sense of control and empowerment [47^▪^].

Furthermore, preliminary data from an ongoing cohort study suggest a possible GD prevalence of approximately 7.0% among individuals with FEP [33]. Early evidence also indicates that GD may be associated with more severe negative symptoms and reduced social and occupational functioning in this population [33,48]. However, the direction of causality remains to be established.

Understanding both the potential harms and benefits of gaming in the context of psychotic disorders underscores the pressing need to explore how individuals themselves perceive and make sense of these experiences. Yet, despite increasing recognition of the clinical relevance of this intersection, no studies to date have captured the *lived experience* of those affected, leaving a critical knowledge gap.

To fully understand the implications of GD in the context of FEP, it is essential to integrate the voices of persons with lived experience (PWLE) in research and clinical interventions. While quantitative studies offer valuable epidemiological and clinical data, they often overlook the subjective dimensions of recovery, such as personal meaning, values, and life aspirations. In contrast, qualitative research provides a unique window through the experience of PWLE with psychosis, as exemplifier by the study of personal recovery from a psychotic disorder. Although there is growing consensus that recovery extends beyond symptom reduction to include personal recovery – defined by the individual's own perception of a satisfying, hopeful, and meaningful life despite ongoing challenges – multiple, and at the time conflicting, conceptualizations of this concept have emerged. A systematic review of qualitative studies focussing on the perspective of PWLE with psychosis led to the development of the CHIME framework (Connectedness, Hope and optimism, Identity, Meaning in life, and Empowerment), which is now a widely accepted model for conceptualizing personal recovery [49] and has reshaped the vision of treatment goals in this population. This example illustrates how qualitative research, could reveal the nuanced experiences of PWLE navigating the intersection of FEP and GD. These insights are crucial for developing tailored interventions and services that truly support the recovery process.

Involving PWLE directly in research teams is another key strategy to improve our understanding of lived experience. Research often neglects the priorities and concerns identified by individuals with lived experience of mental illness [50]. However, engaging PWLE in the development of outcome measures ensure that their perspectives are meaningfully integrated in both the design and interpretation of research.

The goals of the present paper are: to provide a clinical situation illustrating the complex relationship between a psychotic disorder and a gaming disorder that inspired a research program; to offer preliminary observations of a qualitative study describing motivations and consequences of gaming in persons living with FEP; and to provide guidance for future studies exploring the lived experience of GD comorbid to psychotic disorders. 

**Box 1:**
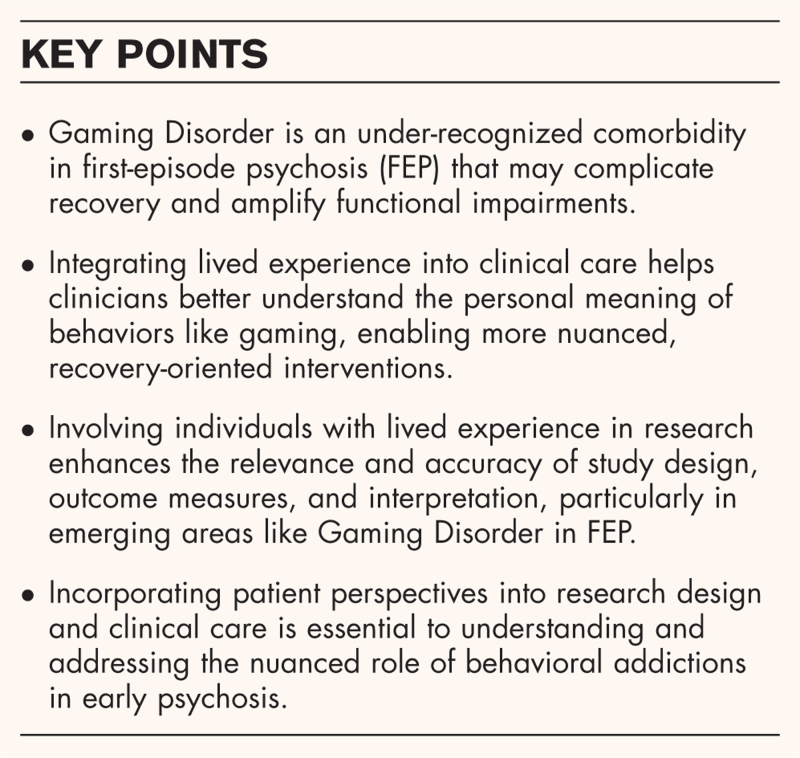
no caption available

### Our first observations of gaming disorders in first-episode psychosis

This section presents a fictional case based on our first few observations of GD in FEP. A young man was admitted to a FEP program with unspecified psychosis following a hospitalization for a FEP. A year postadmission, his positive symptoms had fully remitted with a low-dose antipsychotic that was well tolerated, and with intensive case-management. While he expressed his motivation to go back to work, he procrastinated in applying to jobs offered. He remained socially withdrawn, reporting dysphoric mood without other depressive symptoms and difficulties reconnecting with peers. After a while, his clinician asked about his daily activities, and realized that he was spending most of his time gaming online, an aspect that had not been explored previously.

A detailed clinical interview revealed longstanding, intensive engagement with games such as *League of Legends*, *Guild Wars 2* and *Legends of Runeterra*. Notably, in *League of Legends*, he accumulated over 400 24-h sessions over 8-year and maintained two accounts for 4 years to facilitate progression. From his perspective, gaming had both positive and negative impacts. It provided entertainment and social interaction – primarily through streaming platforms and in-game communities. – but also sustained his social withdrawal and delayed his return to work. His thoughts on gaming's impact on his mental health were complex: he associated it with anxiety and a sense of stagnation in life, though he did not perceive it as a direct trigger for his initial psychotic symptoms. However, he speculated that certain visual hallucinations might have been influenced by in-game experiences. The identification of GD led to a re-interpretation of the patient's negative symptoms and difficulties in functioning that were since then understood as consequences of environmental under-stimulation and GD which thereafter became a major focus of therapeutic interventions.

Such cases catalyzed our research into GD in individuals with FEP, highlighting the need to systematically assess gaming activities in this population, as well as their positive or negative impacts on the recovery process. Furthermore, they raised the possibility that better understanding the links between comorbid GD and psychotic disorders may shed light on obstacles to recovery in these youths [51].

### A mixed-methods exploration of gaming and recovery

Building on these insights, we designed a mixed method study incorporating quantitative and qualitative data focused on lived experiences to explore motivations for gaming and perceived positive and negative consequences among individuals with GD and FEP. This study is being conducted within two FEP programs in the Quebec Province, Canada which provide early intervention for adults aged 18–35 presenting with a FEP [48,52,53].

The quantitative component consists of a prospective cohort study assessing gaming behaviors, clinical data, and psychotic symptoms profiles among all admitted patients. The qualitative component involves individual semi-structured interviews with participants who regularly engage in video gaming. The objective is to explore gaming motivations and the perceived benefits and adverse consequences of gaming within the context of psychosis. The interview guide was co-developed with PWLE of both FEP and GD, to enhance relevance and meaningful interpretation [48,54,55]. This project was approved by the local institution ethic review board. All participants in the semi-structured interviews provided written informed consent. All collected data and cotes were anonymized.

#### Preliminary observations of the qualitative study

Although analyses are ongoing and thematic saturation has not yet been reached, preliminary findings suggest that the role of video games in the lives of individuals with FEP is complex and highly individualized. These early insights underscore the importance of attending to lived experiences to inform more person-centered clinical care and research. In light of the lack of research in this area, selected excerpts from the narratives of four participants are presented to provide a preliminary overview of the data and to enhance understanding of dual diagnosis from the perspective of lived experience. These excerpts are presented and provisionally grouped into two broad categories – motivations and consequences – reflecting the early stage of thematic development.

#### Gaming motivations in individual living with first episode psychosis

Gaming can emerge as default leisure activity, especially in contexts marked by social isolation or lack of structured social engagements:

“Gaming… it's my main hobby. If I don’t have plans or social activities, that's what I’ll do by default.”

Gaming can also emerge for some individual in a motivation for self-improvement, achievement, and skill development. One participant described enjoying challenging gameplay demanding both motor coordination and cognitive effort:

“It's kind of like pushing my limits. Some games… they’re just hard to play. You need strong motor skills. That's the kind of game I’ve always appreciated.”

Beyond the pursuit of skill development, another participant described gaming as a compensatory strategy to cope with perceived cognitive impairments – such as reduced processing speed and attention difficulties –attributed to antipsychotic treatment: *“[video games] help keep my brain active”,* expressing a desire to counteract these effects through sustained mental engagement.

For another, gaming was a valued personal moment for decompression, purposefully reserved for unwinding:

“It's the moment I disconnect. I’m not thinking about anything else. I get through all my tasks, finish everything, and I tell myself, ‘Okay, in an hour I’ll be done, and I’ll finally be able to sit down and play.’ I really look forward to it. It's my moment—something I always keep for myself.”

#### Perceived benefits of gaming in first episode psychosis

While excessive gaming habits are often portrayed as maladaptive, the following examples suggest that gaming could be beneficial for some individuals with FEP.

For some, gaming created meaningful opportunities to maintain relationships and feel connected to others. One participant explained that the value was less in the game itself and more about the shared space it created:

“Sometimes we don’t even play — we just hang out on Discord and talk for an hour or two.”

Another participant, reflecting on long-standing online friendships, described these connections as deeply meaningful:

“There are some people I’ve known for almost six years… it's kind of like family.”

Gaming can also be described as a source of personal achievement, contributing to enhanced self-esteem. One participant emphasized a preferences for games requiring strategy and problem-solving:

“You need to think, reflect, even search online to figure out what you’re doing wrong or how to improve.”

He also highlighted the effort, time, and skill needed to succeed, noting that gaming offered *“a sense of personal accomplishment.”*

These narratives suggest that, for some individuals, gaming can serve as a structured and motivating environment to experience personal challenges and confidence.

Notably, one participant used gaming to manage anxiety linked to emerging psychotic symptoms:

“During my psychotic episodes, gaming helped me stay focused on a single task, allowing me to ignore what I was hearing or seeing. As soon as I sensed the first signs, like dizziness or voices, I would start playing as a preventive measure.”

Participants described a range of motivations and perceived benefits associated with gaming, emphasizing its functional role in daily life – from emotional regulation to cognitive stimulation and symptom management. These insights support a nuanced, person-centered approach to treatment, recognizing gaming's adaptive potential, despite the following negative consequences that were described by participants.

#### Negative consequences of gaming in persons living with first-episode psychosis

A common theme was the disruption of daily routines and difficulty maintaining basic self-care due to prolonged gaming sessions. Participants described episodes of intense procrastination, often prioritizing gaming over essential tasks:

“Sometimes I just keep playing and think, ‘I’ll do that tomorrow, it's no big deal,’ and then I put it off again. I procrastinate a lot. It's not that I forget—I just push everything aside.” “I struggle to take care of myself because of gaming… I have trouble doing everything, like eating at regular times. I just get lazy.”

One participant reported playing for extended periods – sometimes overnight – which significantly interfered with sleep and recovery:

“Friday nights, after work, I’d sit down to play. But instead of playing for two hours, I’d go for twelve. I’d start at 8 p.m. and keep going until 8 a.m.”

Another participant reflected on the consequences of these late-night gaming sessions, expressing guilt and self-awareness about their impact:

“When I did that—going to bed at 3 a.m. and having to get up for work the next day—I definitely felt guilty. I knew I wouldn’t be in good shape for the day ahead. I’ve always known it was irresponsible.”

Some participants expressed concerns about excessive gaming impact on physical health: “I can’t spend my whole life gaming. That's not really a life – just gaming all the time.” “Because of my weight… I don’t move when I game. I can sit for hours. I’ve gained a lot of weight since my psychosis because I’ve been gaming so much. I need to stop – otherwise, I’ll end up dying from this.”

Gaming was also reported to interfere with medication adherence and appointment attendance, particularly when it absorbed the participant's attention and disrupted routines: *“It's happened a lot. I’ve forgotten appointments or meds because I was gaming. Honestly, if my mom didn’t remind me, I’d forget everything.”*

For one individual video game was a form of maladaptive coping mechanism used to avoid confronting personal or emotional difficulties. He described it as a vicious cycle where avoidance led to worsening psychosocial outcomes:

“If you keep numbing yourself with [video games], your problems don’t get better. You go to bed later, you’re more tired, more irritable, more likely to argue. You end up with more social problems. I’ve used gaming like a drug—it became part of the problem.”

## DISCUSSION AND CONCLUSION

The relationship between GD and psychotic disorders is complex and multifaceted. Shared features such as social withdrawal and lack of motivation are core elements of both conditions, complicating efforts to disentangle their interplay. Preliminary data suggest that individuals with comorbid GD and FEP may experience more pronounced negative symptoms and greater impairments in social and occupational functioning compared to those with FEP alone [33]. While these associations are clinically important, their directionality remains unclear. This further underscores the importance of focussing on lived experiences to better understand how these conditions intersect from a personal recovery perspective.

These findings point to the potential value of developing targeted interventions to mitigate the adverse effects of GD in this population. However, it is essential that these interventions be informed by the lived experience of those affected. Preliminary examples from our qualitative research reveal a wide range of motivations for gaming, along with both perceived benefits and negative consequences. These first-person accounts highlight the functional role gaming may play – from coping with symptoms and regulating anxiety to fostering social connection and cognitive engagement.

Clinically, these insights support the development of patient-centered therapeutic approaches that acknowledge the role of gaming in daily life. Rather than promoting strict abstinence, treatments approach should aim to improve self-regulation of gaming behaviors [56]. A harm-reduction framework should also be considered, encouraging less harmful pattern of gaming or alternative strategies that serve similar functions, as seen in evidence-based approaches for SUD [51]. Recognizing the perceived positive and negative consequences of gaming may also serve as a meaningful starting point for behavior change in individuals seeking to reduce problematic use without discontinuing gaming entirely. Understanding the personal experience of gaming can also offer valuable insight into individual needs and strengths.

These findings underscore the critical value of integrating lived experience into psychiatric research and care. Exploring first-person narratives not only addresses gaps left by traditional empirical approaches but also fosters a more holistic understanding of the phenomenon. Such an approach can guide the development of tailored screening tools and intervention strategies that are better aligned with patient values and recovery goals.

As research in this area continues to evolve, support from funding agencies will be pivotal in ensuring the meaningful inclusion of PWLE, not only in research design but also in clinical education and training. Embedding this perspective into both practice and pedagogy is fundamental to advancing recovery-oriented care in the context of emerging and complex conditions such as GD and FEP.

## Acknowledgements

*We thank all participants in our study, as well as the patient partners whose contributions were essential to this work.*

### Financial support and sponsorship

*M.H.L. is supported by the Frederick Banting and Charles Best Canada Graduate Scholarship Doctoral Awards from the Canadian Institute of Health Research (FID-172598) and by Mitacs Grant (IT34510). The funders played no role in the redaction of this article, and we have no restrictions regarding the submission of this manuscript for publication.*

### Conflicts of interest

*There are no conflicts of interest.*

## REFERENCES AND RECOMMENDED READING

Papers of particular interest, published within the annual period of review, have been highlighted as:

▪ of special interest
▪▪ of outstanding interest
